# Obstacle-aware inverse kinematics of variable-length continuum robots via teaching–learning-based optimization with experimental validation

**DOI:** 10.1038/s41598-026-46132-6

**Published:** 2026-04-01

**Authors:** Abdelhamid Ghoul, Awais Muhammad Sattar, Mohammed Amin Adoul, Osama Sadki, Selman Djeffal

**Affiliations:** 1 Automatic Control, Department of Computer Science, Electrical and Space Engineering, Luleå University of Technology, 97187 Luleå, Sweden; 2 Operation and Maintenance, Department of Civil, Environmental and Natural Resources Engineering, Luleå University of Technology, 97187 Luleå, Sweden; 3 Department of Mechanical Engineering, National Polytechnic School, 25000 Constantine, Algeria

**Keywords:** Continuum robots, Variable-length continuum robot, Inverse kinematics, Constrained optimization, Obstacle avoidance, Teaching–learning-based optimization (TLBO), Trajectory tracking, Engineering, Mathematics and computing, Physics

## Abstract

Continuum robots offer high dexterity and compliance, which makes them attractive for tasks in confined, hazardous, and hard-to-reach environments. Despite this potential, inverse kinematics (IK) for multi-section continuum robots remains challenging due to strong nonlinearities and redundancy, and the problem becomes more demanding when each section can actively change its backbone length. This paper addresses obstacle-aware IK for a cable-driven variable-length continuum robot by formulating IK as a constrained optimization problem built on a constant-curvature forward kinematic model. A teaching–learning-based optimization (TLBO) algorithm is adopted to search for section bending angles, orientation angles, and section lengths that minimize end-effector tracking error while avoiding static obstacles through a capsule-based penalty constraint handling strategy that accounts for the robot’s physical radial dimension. The approach is evaluated through multiple three-dimensional MATLAB simulations, including linear and circular trajectory tracking with and without obstacle avoidance, and is benchmarked against particle swarm optimization (PSO), a real-coded genetic algorithm (GA), and differential evolution (DE) over 30 independent runs. Statistical analysis shows that TLBO achieves the best or near-best tracking accuracy (mean error $7.84\times 10^{-5}$ mm, best $4.95\times 10^{-7}$ mm) while requiring no algorithm-specific tuning parameters. The method is further validated experimentally on a Continuum Bionic Handling Assistant (CBHA) platform by comparing the IK-derived cable-length profiles with potentiometer-based measurements. The results demonstrate accurate trajectory tracking in simulation and good agreement with experimental cable-length measurements, supporting the feasibility of TLBO for constrained IK of variable-length continuum robots.

## Introduction

Continuum robots have attracted considerable interest for tasks that require safe interaction with complex environments, such as minimally invasive surgery, search-and-rescue in collapsed structures, and inspection in hazardous sites (e.g., nuclear facilities)^1–6^. Their intrinsic compliance and high dexterity, inspired by biological trunks and tentacles, enable navigation through confined and cluttered spaces where rigid-link manipulators may struggle^7–10^. Achieving reliable motion planning and control for such systems, however, depends critically on accurate kinematic models.

Inverse kinematics (IK) of continuum robots remains challenging because the mapping from actuation to end-effector pose is highly nonlinear and typically redundant. While forward kinematic models (FKMs) can often be derived under simplifying assumptions, closed-form inverse kinematic models (IKMs) are generally not available for practical multi-section designs. A widely adopted hypothesis is the constant-curvature (CC) assumption^10–16^, in which each robot section is approximated by a circular arc, leading to compact and computationally convenient FKMs. Alternative variable-curvature (VC) descriptions represent each section as a sequence of circular arcs, which can better capture complex shapes at the cost of increased model complexity^17–20^. Despite these developments in FKMs, solving IK remains difficult because of the nonlinear equations involved and the existence of multiple feasible configurations for a single target pose.

To address these difficulties, several approaches have been explored. Optimization-based methods leverage FKMs to search for configurations that minimize a tracking error without requiring closed-form inverse solutions. Learning-based approaches have also been proposed to approximate IK mappings from data^21–23^. For example, the IKM of a dual-backbone continuum robot was addressed using a pseudo-rigid-body model and a neural approximation derived from the forward model^22^. Feed-forward neural networks have been used to relate end-effector motion to cable forces in cable-driven systems^23^, and multi-layer neural models have been reported for approximate IK solutions^24^. Supervised learning frameworks have further been investigated to learn IK from generated datasets without detailed analytical modeling^25,26^. Although data-driven methods can achieve good accuracy within the training domain, incorporating explicit constraints such as collision avoidance and ensuring consistent behavior outside the training distribution remain open challenges.

Obstacle avoidance is a critical requirement for continuum robots operating in cluttered environments. Several strategies have been explored in the literature. Potential-field methods, originally introduced for rigid manipulators^29^, have been adapted for continuum robots by distributing repulsive fields along the robot backbone^30,31^. Sampling-based planners such as rapidly-exploring random trees (RRT) have been combined with continuum kinematics for collision-free path planning^32^. Jacobian-based methods handle obstacle constraints via inequality-constrained optimization at each time step^33^. More recently, reinforcement learning has been applied to learn collision-avoidance policies directly from simulated interactions^34^. However, potential-field approaches can suffer from local minima, sampling-based planners may struggle with narrow passages, and learning-based methods require extensive training data and may not generalize to unseen obstacle configurations. In contrast, the penalty-based formulation adopted in this work integrates obstacle avoidance directly into the IK objective function, avoiding local-minimum traps through the global search capability of the metaheuristic optimizer, and does not require training data.

An additional layer of difficulty arises for variable-length (VL) continuum robots, where the backbone length of each section can actively change. Length actuation can significantly enlarge the reachable workspace compared with constant-length designs, but it also increases the dimensionality of the IK problem and may amplify redundancy. Despite the practical relevance of VL robots, relatively few studies have addressed IK for multi-section continuum robots with extensible sections under task constraints.

In this paper, we formulate obstacle-aware IK for a cable-driven, variable-length, multi-section continuum robot as a constrained optimization problem based on a CC forward kinematic model. We solve the resulting problem using teaching–learning-based optimization (TLBO)^27,28^, which reduces reliance on algorithm-specific tuning parameters commonly required in other metaheuristics. To validate this claim, we present a comprehensive quantitative comparison with particle swarm optimization (PSO), a real-coded genetic algorithm (GA), and differential evolution (DE), all evaluated under identical conditions over 30 independent runs with full statistical analysis. The proposed framework is evaluated in multiple three-dimensional simulation studies for trajectory tracking with and without obstacle avoidance, and it is further validated experimentally on a Continuum Bionic Handling Assistant (CBHA) platform by comparing IK-derived cable-length profiles with potentiometer-based measurements.

Contributions. The main contributions of this work are:

- A constrained optimization formulation of IK for a cable-driven variable-length continuum robot based on a constant-curvature forward kinematic model.
- A capsule-based obstacle-avoidance mechanism integrated into the IK objective using an explicit penalty formulation that accounts for the robot’s physical radial dimension.
- A TLBO-based IK solver evaluated in three-dimensional simulations for linear and circular trajectory tracking, including obstacle-aware scenarios.
- A comprehensive quantitative benchmarking of TLBO against PSO, GA, and DE with convergence analysis, computational cost comparison, and statistical testing over 30 independent runs.
- A warm-start seeding strategy with ablation study demonstrating improved convergence for sequential trajectory tracking.
- Experimental validation on a CBHA-class continuum robot through comparison of computed and measured cable-length profiles.

## Robot description and forward kinematics

### Robot architecture

**Fig. 1:**
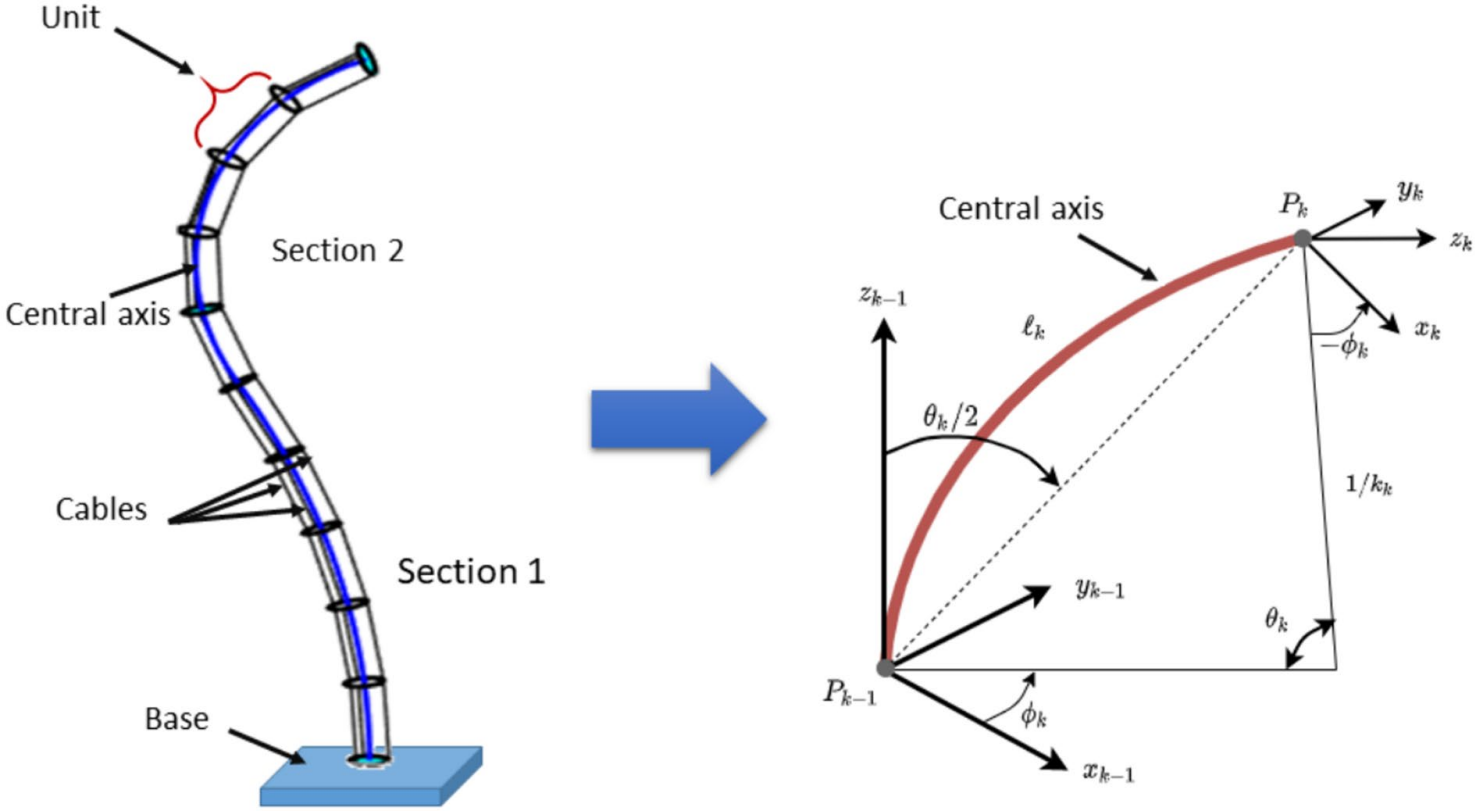
(Left) Description of the considered continuum robot. (Right) Detailed description of the centerline in each section under the adopted kinematic assumptions.

The continuum robot considered in this study (Fig. 1) is a cable-driven, trunk-like manipulator composed of a flexible cylindrical backbone and a set of spacer disks of identical diameter. Each disk is actuated by three tendons uniformly distributed at $120^\circ$ around the backbone. The robot is organized into sections (indexed by *k*), and each section consists of a serial chain of identical units (Fig. 1).

Under the constant-curvature modeling assumption, each *section* is characterized by a bending angle $\theta _k$, an orientation angle $\phi _k$, and a backbone length $\ell _k$. A *unit* is the physical segment between two consecutive disks within a section. All units within the same section share the same curvature $\kappa _k = \theta _k / \ell _k$; therefore, increasing the number of units $m_k$ within a section increases the physical granularity and the number of cable attachment points (improving the constant-curvature approximation and structural rigidity), but does *not* introduce additional kinematic variables. The section backbone length $\ell _k$ is distributed equally among its $m_k$ units.

In this work, we focus on a variable-length (VL) continuum robot in which the backbone length of each section can extend or contract depending on the task requirements. Depending on the design, the backbone can be virtual (e.g., reconstructed from geometry) as in^15,18,21^, or physically realized as a structural element as in^3^. The main geometric parameters and reference frames used for modeling are illustrated in Fig. 1.

### Constant-curvature forward kinematics

Based on Fig. 1 and the constant-curvature assumption commonly adopted for continuum robots^13^, the end-effector pose is obtained by composing the homogeneous transformations of all sections:

1 $$\begin{aligned} \textbf{T}_n^{0}= \prod _n^{0}{\textbf{T}_{k}^{k-1}} \end{aligned}$$

where the transformation of section *k* with respect to section $k-1$ is

2 $$\begin{aligned} \textbf{T}_{k}^{k-1}= \left( \begin{array}{c|c} \textbf{R}_{k}^{k-1} & \textbf{P}_{k}^{k-1} \\ \hline \textbf{0}_{1\times 3} & 1 \end{array}\right) . \end{aligned}$$

Here, $\textbf{R}_{k}^{k-1}$ is the rotation matrix and $\textbf{P}_{k}^{k-1}$ is the translation vector. Under the arc-parameter representation, they are given by

3 $$\begin{aligned} \begin{aligned} \textbf{R}_{k}^{k-1}&= \textbf{rot}{(Z_{k-1},\phi _k)}\cdot \textbf{rot}{(Y_{k-1},\theta _{k})}\cdot \textbf{rot}{(Z_{{k-1}},-\phi _k)} \\ &=\begin{bmatrix} c^2\phi _k c\theta _{k}+s^2\phi _k & c\phi _k s\phi _k c\theta _{k} -c\phi _k s\phi _k \quad \quad & c\phi _k s\theta _{k} \\ c\phi _k s\phi _k c\theta _{k} -c\phi _k s\phi & s^2\phi c\theta _{k} +c^2\phi _k & s\phi _k s\theta _{k} \\ -c\phi _k s\theta _{k} & -s\phi _k s\theta _{k} & c\theta _{k} \end{bmatrix} \end{aligned} \end{aligned}$$

and

4 $$\begin{aligned} \textbf{P}_{k}^{k-1} = \left\{ \begin{array}{ll} \frac{\ell _{k}}{ \theta _{k} }(1 - \cos (\theta _{k}))\cos (\phi _k) \\ \frac{\ell _{k}}{ \theta _{k} }(1 - \cos (\theta _{k}))\sin (\phi _k) \\ \frac{\ell _{k}}{ \theta _{k} } \sin (\theta _{k}) \end{array} \right. \end{aligned}$$

where $\ell _k$ is the backbone length of section *k*. The variables and parameters involved in (3) and (4) are summarized in Table 1.

### Robot parameters used in this study

Unless otherwise stated, we consider a two-section robot ($n=2$). The backbone length of each section ranges from 100 mm (fully contracted) to 300 mm (fully extended), resulting in a total backbone length between 200 mm and 600 mm. Each section is modeled with five units and identical disk radius. The corresponding parameters are listed in Table 1.

**Table 1:** Parameters of the considered flexible continuum robot.

	Section 1	Section 2	Description
${m_k}$	5 units	5 units	Number of units
${\ell _{\min ,k}}$	100 mm	100 mm	Minimum contraction length in each section
${\ell _{\max ,k}}$	300 mm	300 mm	Maximum extension length in each section
${r_{k}}$	12 mm	12 mm	Radius of disks

## Workspace generation

**Fig. 2:**
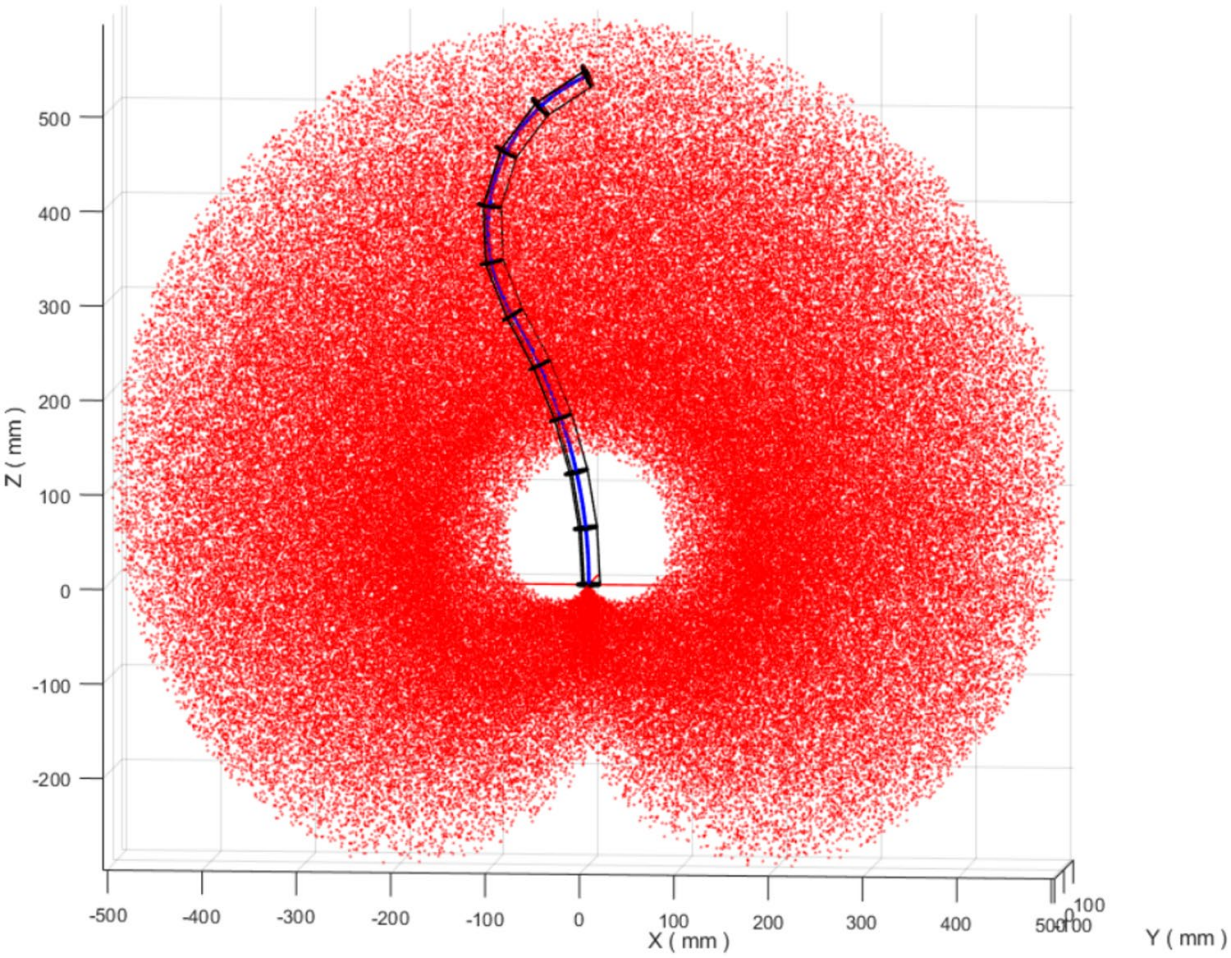
2D workspace for a VL two sections continuum robot.

Workspace characterization provides a first-order view of the set of end-effector positions reachable under the adopted kinematic model. In this study, the workspace is generated by sampling the forward kinematics over the section bending and orientation angles. Specifically, the bending angles are varied within $\theta _{1,2}\in [-\pi ,\pi ]$ and the orientation angles within $\phi _{1,2}\in [0,\pi ]$, while respecting the section-length limits listed in Table 1. The resulting reachable region for the two-section variable-length (VL) robot is shown in Fig. 2.

**Fig. 3:**
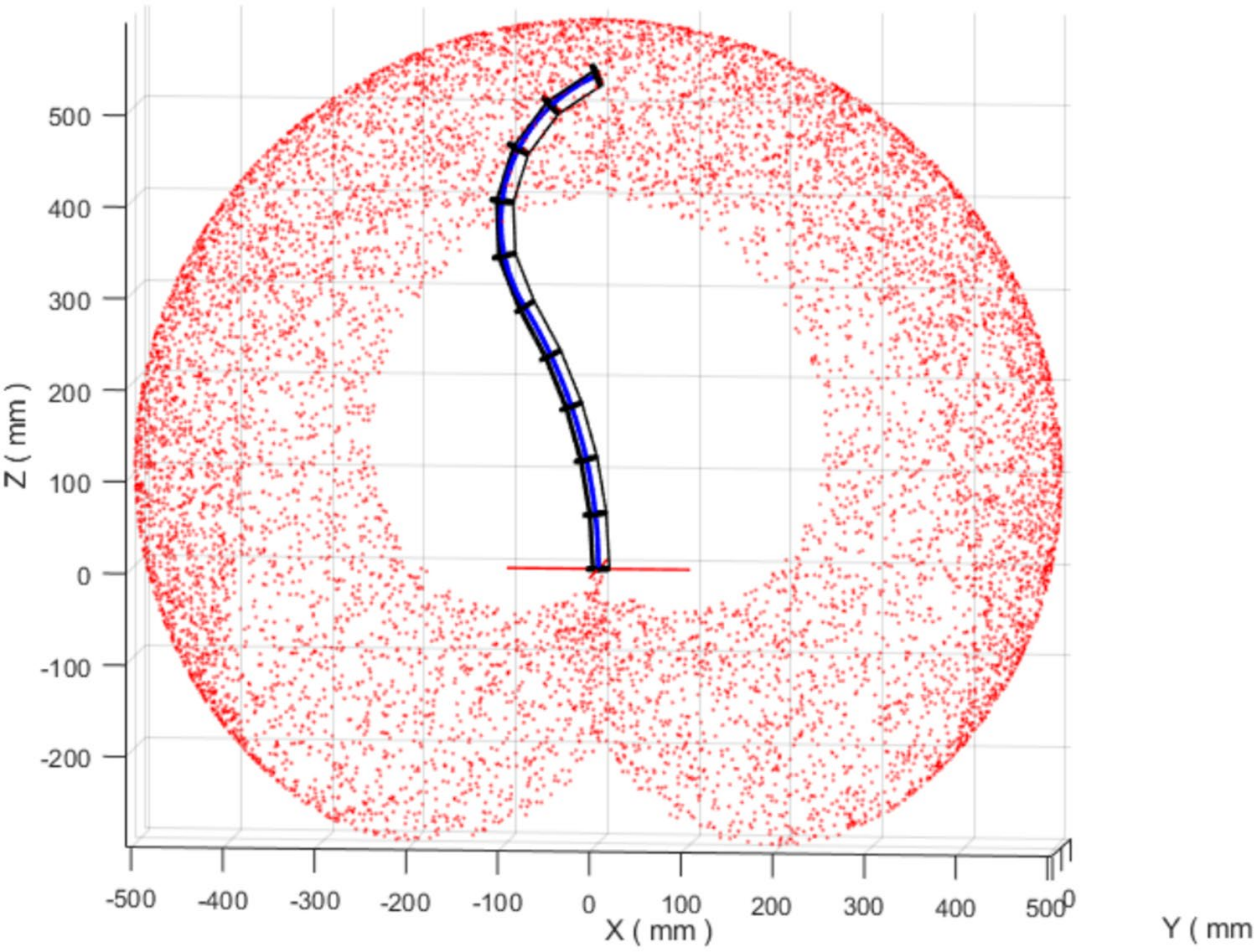
2D workspace for a CL two sections continuum robot ($\ell _1=\ell _2=300$ mm).

For comparison, Fig. 3 shows the workspace obtained for an equivalent two-section constant-length (CL) robot with fixed backbone lengths $\ell _1 = \ell _2 = 300$ mm (the maximum extension from Table 1). Allowing the backbone length to vary enlarges the reachable set and improves coverage of the interior region of the workspace. In particular, the VL robot can reach configurations that are not accessible under fixed-length actuation, which is consistent with the intuitive effect of extending and contracting the backbone to adapt the robot posture.

## Teaching–learning-based optimization (TLBO)

Teaching–learning-based optimization (TLBO) is a population-based metaheuristic introduced by Rao et al.^27^ and further developed in^28^. TLBO iteratively improves a population of candidate solutions through two successive stages: (i) a *teacher phase*, which shifts the population toward the best current solution, and (ii) a *learner phase*, which promotes improvement through pairwise interactions between solutions. In the present work, each candidate solution encodes the kinematic decision variables (e.g., $\theta _k$, $\phi _k$, and $\ell _k$), and its quality is evaluated via the objective function defined in Section "Objective function and problem formulation".

Let *n* denote the population size (number of learners) and *m* the number of decision variables (topics). We denote the value of variable *j* for candidate *k* at iteration *i* by $X^{i}_{j,k}$. Since the IK problem is posed as a minimization task, lower fitness values indicate better solutions.

### Teacher phase

In the teacher phase, the current best candidate (teacher) guides the population by attempting to improve the mean value of each variable across the population. The population mean for variable *j* at iteration *i* is

5 $$\begin{aligned} M^\mathrm i_{\mathrm j}=\frac{\sum \limits _{\mathrm k = 1}^n{X^\mathrm i_{\mathrm j,\mathrm k}} }{n} \end{aligned}$$

Let $X^\mathrm i_{\mathrm j,\mathrm {k_{best}}}$ denote the teacher solution (the best candidate for the current iteration). The mean-shift term is computed as

6 $$\begin{aligned} d^\mathrm i_{\mathrm j,\mathrm k}=r\left( X^\mathrm i_{\mathrm {j,k_{best}}}-T_FM^\mathrm i_\mathrm j )\right. \end{aligned}$$

where $r\in [0,1]$ is a random number and $T_F\in \{1,2\}$ is the teaching factor. Candidate solutions are then updated as

7 $$\begin{aligned} Xnew^\mathrm i_{\mathrm j,\mathrm k}=X^\mathrm i_{\mathrm j,\mathrm k}+d^\mathrm i_{\mathrm j,\mathrm k} \end{aligned}$$

After evaluating the objective function for the updated candidates, a greedy selection step retains the better solution between $X^\mathrm i_{\mathrm j,\mathrm k}$ and $Xnew^\mathrm i_{\mathrm j,\mathrm k}$.

### Learner phase

In the learner phase, candidates improve through interaction with other randomly selected candidates. Two candidates *A* and *B* are selected at random and updated according to their fitness values:

8 $$\begin{aligned} \left\{ {\begin{array}{*{20}{c}}{Xnew^\mathrm i_{\mathrm j,A}=X^\mathrm i_{\mathrm j,A}+r \left( X^\mathrm i_{\mathrm j,A}-X^\mathrm i_{\mathrm j,B} )\right. \quad if \quad F^\mathrm i_A<F^\mathrm i_B }\\ {Xnew^\mathrm i_{\mathrm j,A}=X^\mathrm i_{\mathrm j,A}+r \left( X^\mathrm i_{\mathrm j,B}-X^\mathrm i_{\mathrm j,A} )\right. \quad if \quad F^\mathrm i_B<F^\mathrm i_A }\end{array}} \right. \end{aligned}$$

where $F^i_A$ and $F^i_B$ denote the fitness values of candidates *A* and *B*, respectively. As in the teacher phase, a greedy selection step is applied after evaluating the updated solutions. Equations (6)–(8) are used here in the minimization setting.

### Comparison with other metaheuristics

Many metaheuristic optimizers require algorithm-specific hyperparameters (e.g., crossover and mutation rates in genetic algorithms or acceleration constants in particle swarm optimization), which can influence performance and require tuning. In contrast, TLBO requires only the population size and the stopping criterion as control parameters—no algorithm-specific parameters need to be set. Table 2 summarizes the typical parameters required by several common metaheuristics, including the four algorithms benchmarked in this study.

**Table 2:** The required algorithm-specific parameters for each metaheuristic.

Algorithm	Required algorithm-specific parameters
Genetic algorithm (GA)	Crossover probability, mutation rate, selection method.
Particle swarm optimization (PSO)	Inertia weight, acceleration constants ($c_1$, $c_2$), max. velocity.
Differential evolution (DE)	Scaling factor (*F*), crossover rate (*CR*).
Artificial bee colony (ABC)	Onlooker bees number, employed bees number, food sources.
Harmony search (HS)	Range of each variable, pitch rate, number of improvisations.
Teaching–learning-based optimization (TLBO)	None (only population size and stopping criterion).

### Algorithmic complexity

Each iteration of TLBO consists of two phases (teacher and learner), each requiring $\mathcal {O}(N_p)$ objective function evaluations, where $N_p$ is the population size. Each objective function evaluation involves one forward kinematics computation (trigonometric operations for each section) and $N_b$ distance computations for capsule-based collision checking. Therefore, the total computational cost per trajectory point is $\mathcal {O}(2 \cdot N_p \cdot k_{\max } \cdot N_b)$, where $k_{\max }$ is the maximum number of iterations. For our two-section robot with $N_p=40$, $k_{\max }=2000$, and $N_b=10$, this amounts to approximately $1.6 \times 10^6$ distance computations per trajectory point, completing in approximately 0.27 s on an Intel Core i7-1355U processor with 32 GB RAM running MATLAB R2025b under Windows 11.

## Objective function and problem formulation

Inverse kinematics (IK) is formulated here as a constrained optimization problem. For each target point on a prescribed trajectory, we seek the robot configuration that minimizes the end-effector tracking error while satisfying feasibility constraints, including collision avoidance and admissible joint/length limits. The decision variables are the kinematic parameters of all sections, i.e., the bending angles, orientation angles, and (for the variable-length robot) the section backbone lengths. For a two-section robot, this can be written as $\textbf{q} = (\theta _1,\phi _1,\ell _1,\theta _2,\phi _2,\ell _2)$, with lower and upper bounds $\textbf{q}_{\min }$ and $\textbf{q}_{\max }$ defined by the physical limits of the robot (Table 1) and by the admissible angular ranges used in the workspace analysis.

### Tracking objective

Let $(X_{c_i},Y_{c_i},Z_{c_i})$ denote the Cartesian coordinates of the *i*-th point on the desired trajectory, and let $(P_{X_i},P_{Y_i},P_{Z_i})$ be the end-effector position computed from the forward kinematics for a candidate configuration $\textbf{q}$. The tracking objective is defined as the Euclidean distance between the target point and the end-effector position:

9 $$\begin{aligned} {F_{\text {track}}}= \sqrt{(P_{X_i}-X_{c_i})^2+(P_{Y_i}-Y_{c_i})^2+(P_{Z_i}-Z_{c_i})^2} \end{aligned}$$

### Capsule-based collision-avoidance constraint

**Fig. 4:**
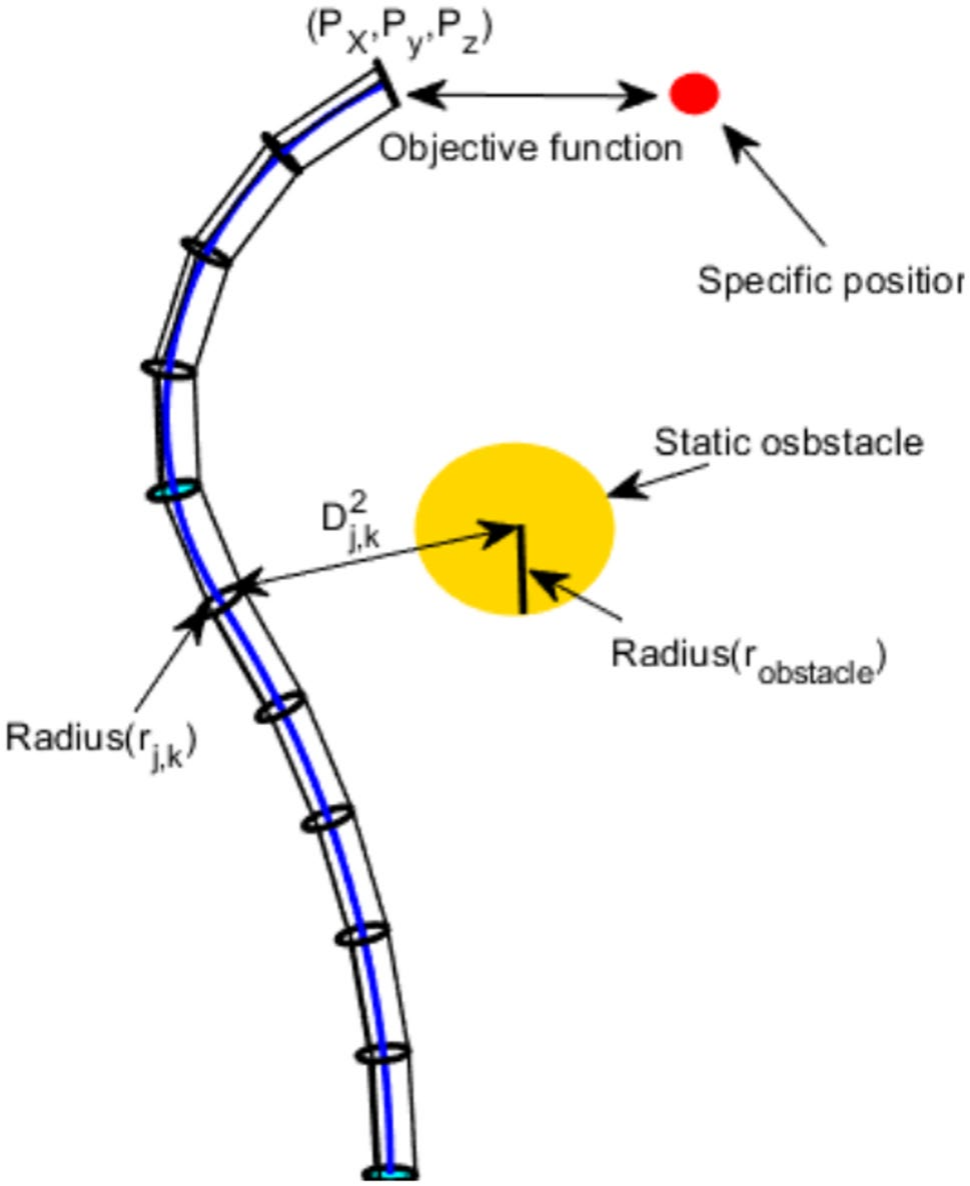
Global view of the governing objective function and the adopted capsule-based strategy for obstacle avoidance. The safety margin is $r_{\text {obs}} + r_{\text {disk}}$..

To incorporate obstacle avoidance, we consider a spherical obstacle with center $\textbf{p}_{\text {obs}}$ and radius $r_{\text {obs}}$ (Fig. 4). Unlike prior formulations that only check the distance between the obstacle center and the robot centerline, we adopt a *capsule-based* collision model that accounts for the robot’s physical radial dimension. For each backbone point $\textbf{p}_b$ (sampled at $N_b$ locations along the robot, with 5 points per section), the Euclidean distance to the obstacle center is

10 $$\begin{aligned} { d_b = \Vert \textbf{p}_b - \textbf{p}_{\text {obs}} \Vert } \end{aligned}$$

Collision avoidance requires that the distance exceeds the sum of the obstacle radius and the robot’s disk radius $r_{\text {disk}}$:

11 $$\begin{aligned} {d_b > r_{\text {obs}} + r_{\text {disk}}, \quad \forall \; b = 1, \ldots , N_b} \end{aligned}$$

This capsule-based formulation prevents physical scraping in narrow spaces, which would be missed by a pure centerline-to-center distance check.

### Explicit penalty formulation

The collision-avoidance constraint (11) is incorporated into the objective via a quadratic penalty function. The total penalized fitness for a candidate configuration $\textbf{q}$ is

12 $$\begin{aligned} { F_{\text {total}}(\textbf{q}) = F_{\text {track}}(\textbf{q}) + P_{\text {obs}}(\textbf{q}) } \end{aligned}$$

where the obstacle penalty is

13 $$\begin{aligned} { P_{\text {obs}}(\textbf{q}) = C \sum _{b=1}^{N_b} \left[ \max \!\left( 0,\; \frac{r_{\text {obs}} + r_{\text {disk}} - d_b}{r_{\text {obs}} + r_{\text {disk}}} \right) \right] ^2 } \end{aligned}$$

Here, $C = 10^6$ is the penalty coefficient, chosen to be several orders of magnitude larger than the maximum expected tracking error ($\approx$600 mm in the worst case within our workspace), ensuring that any constraint violation dominates the fitness and makes infeasible solutions uncompetitive during selection. The quadratic form provides a smooth gradient near the safety boundary while heavily penalizing deep penetrations. The normalized violation term $(r_{\text {obs}} + r_{\text {disk}} - d_b)/(r_{\text {obs}} + r_{\text {disk}})$ ensures scale invariance with respect to obstacle size.

The penalty coefficient *C* is fixed (not adaptive). We verified that values of *C* in the range $[10^4, 10^8]$ produce identical final solutions for our problem, confirming that the specific value is not sensitive as long as it is sufficiently large relative to the tracking error magnitude.

In addition to the obstacle penalty, bound constraints on $\textbf{q}$ are enforced by projecting (clipping) any out-of-range variable back to its admissible interval $[\textbf{q}_{\min }, \textbf{q}_{\max }]$.

### Constrained optimization statement

For each trajectory point, the IK problem can be stated as:

$$\min _{\textbf{q}} \; {F_{\text {total}}(\textbf{q})} \;\; \text {subject to} \;\; \textbf{q}_{\min }\le \textbf{q}\le \textbf{q}_{\max }$$

where the collision-avoidance constraint is absorbed into the objective through the penalty term (13).

Failure cases. When the target point lies inside the obstacle or when the passage is narrower than $2r_{\text {disk}}$, no feasible collision-free solution exists. In such cases, TLBO returns the candidate with the minimum total fitness $F_{\text {total}}$, which will have a large penalty component. The user can detect infeasibility by checking whether $F_{\text {total}} > C$; if so, the returned solution violates the collision constraint and should be flagged for replanning.

Since TLBO is a metaheuristic optimizer that naturally handles unconstrained objectives, the collision-avoidance constraint is incorporated via the penalty-based strategy defined in Section "Explicit penalty formulation". This allows TLBO to solve the IK problem while accounting for obstacle avoidance without requiring gradient information.

## TLBO implementation to solve the IK of a variable-length continuum robot

This section describes how TLBO is used to solve the inverse kinematics (IK) problem formulated in Section "Objective function and problem formulation". The key idea is to solve IK as a sequence of constrained optimization problems, one for each target point along a prescribed trajectory. For each target point, TLBO searches for a feasible robot configuration that minimizes the tracking error while satisfying physical bounds and obstacle-avoidance constraints.

### Decision variables and bounds

For an *n*-section variable-length continuum robot, the optimization vector is defined as

$$\textbf{q}=\left( \theta _1,\phi _1,\ell _1,\;\theta _2,\phi _2,\ell _2,\;\ldots ,\;\theta _n,\phi _n,\ell _n\right) ,$$

where $\theta _k$ and $\phi _k$ are the bending and orientation angles of section *k*, and $\ell _k$ is its backbone length. Each variable is constrained by admissible bounds:

$$\textbf{q}_{\min }\le \textbf{q}\le \textbf{q}_{\max },$$

where length bounds are taken from Table 1 (and Table 4 for the three-section case), and angular bounds follow the limits used in the workspace analysis.

### Trajectory tracking as a sequence of IK problems

Let $\textbf{p}_i=\left[ X_{c_i},Y_{c_i},Z_{c_i}\right] ^\top$ denote the *i*-th point on the desired Cartesian trajectory. For each $\textbf{p}_i$, TLBO computes a corresponding configuration $\textbf{q}_i^\star$ by minimizing the tracking objective (9) and enforcing collision avoidance (11). In practice, trajectory tracking is implemented by repeating the IK optimization for all points $\textbf{p}_i$ along the path.

### Warm-start seeding strategy

To improve numerical stability and encourage smooth configuration changes along the trajectory, the best solution found for $\textbf{p}_{i-1}$ is used to warm-start the search for $\textbf{p}_i$. Specifically, exactly one candidate in the population is seeded with the previous best configuration $\textbf{q}_{i-1}^\star$, while the remaining $N_p - 1$ candidates are randomly initialized within $[\textbf{q}_{\min }, \textbf{q}_{\max }]$. This ratio (1 out of $N_p = 40$, i.e., 2.5% seeded) preserves the global exploration capability of TLBO while providing a warm starting point near the expected solution.

The warm-start strategy also serves as an *implicit redundancy resolution mechanism*: by seeding the population with the previous solution, TLBO naturally tracks a smooth configuration path in joint space, avoiding discontinuous jumps between different IK solution branches. This is analogous to selecting the nearest-neighbor solution in configuration space, but achieved without explicit redundancy resolution constraints.

An ablation study comparing warm-start and cold-start TLBO is presented in Section "Warm-start ablation study".

### Constraint handling via a penalty strategy

TLBO is used here as a derivative-free optimizer. Inequality constraints are handled through the penalty-based strategy defined in Section "Explicit penalty formulation": candidate configurations that violate the obstacle-avoidance margin are assigned a larger fitness value via the penalty term (13), so that feasible solutions are naturally preferred during selection. Bound constraints are enforced by clipping.

### TLBO-based IK procedure

Algorithm 1 summarizes the TLBO-based IK solver applied to a single trajectory point. The objective function evaluation uses the forward kinematics in Section 2 to compute $(P_{X_i},P_{Y_i},P_{Z_i})$ and then evaluates the penalized tracking error (12). The teacher and learner updates follow the TLBO rules (Eqs. (5)–(8)), with greedy selection after each phase.

**Algorithm 1:**
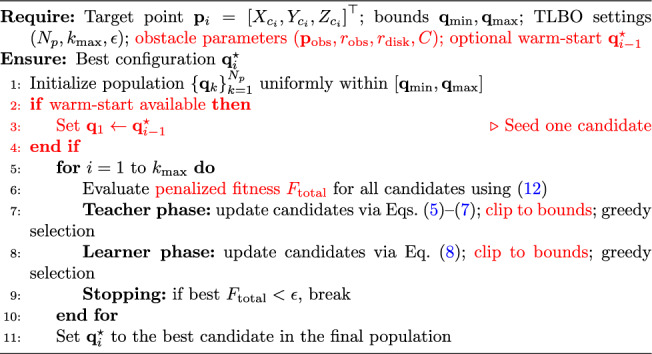
TLBO-based IK for one trajectory point

### Evaluation scenarios

The proposed IK solver is evaluated in simulation for multiple scenarios (linear and circular trajectories, with and without obstacle avoidance), benchmarked against PSO, GA, and DE (Section "Quantitative comparison with other metaheuristic algorithms"), and further validated experimentally on a CBHA-class continuum manipulator (Section "Experimental validation on a CBHA-class continuum robot").

### Variable-length continuum robot following a linear trajectory

We first evaluate the proposed TLBO-based IK solver on a two-section variable-length continuum robot tracking a straight-line trajectory. The desired Cartesian path is defined by

14 $$\begin{aligned} {\left\{ \begin{array}{ll} {X = 10t}\\ {Y = 0}\\ {Z = 300} \end{array}\right. } \end{aligned}$$

with $t = 0:0.1:10$ (101 waypoints). For each target point along the trajectory, TLBO searches for a configuration vector $\textbf{q}=(\theta _1,\phi _1,\ell _1,\theta _2,\phi _2,\ell _2)$ that minimizes the tracking error (9) within the admissible bounds.

**Fig. 5:**
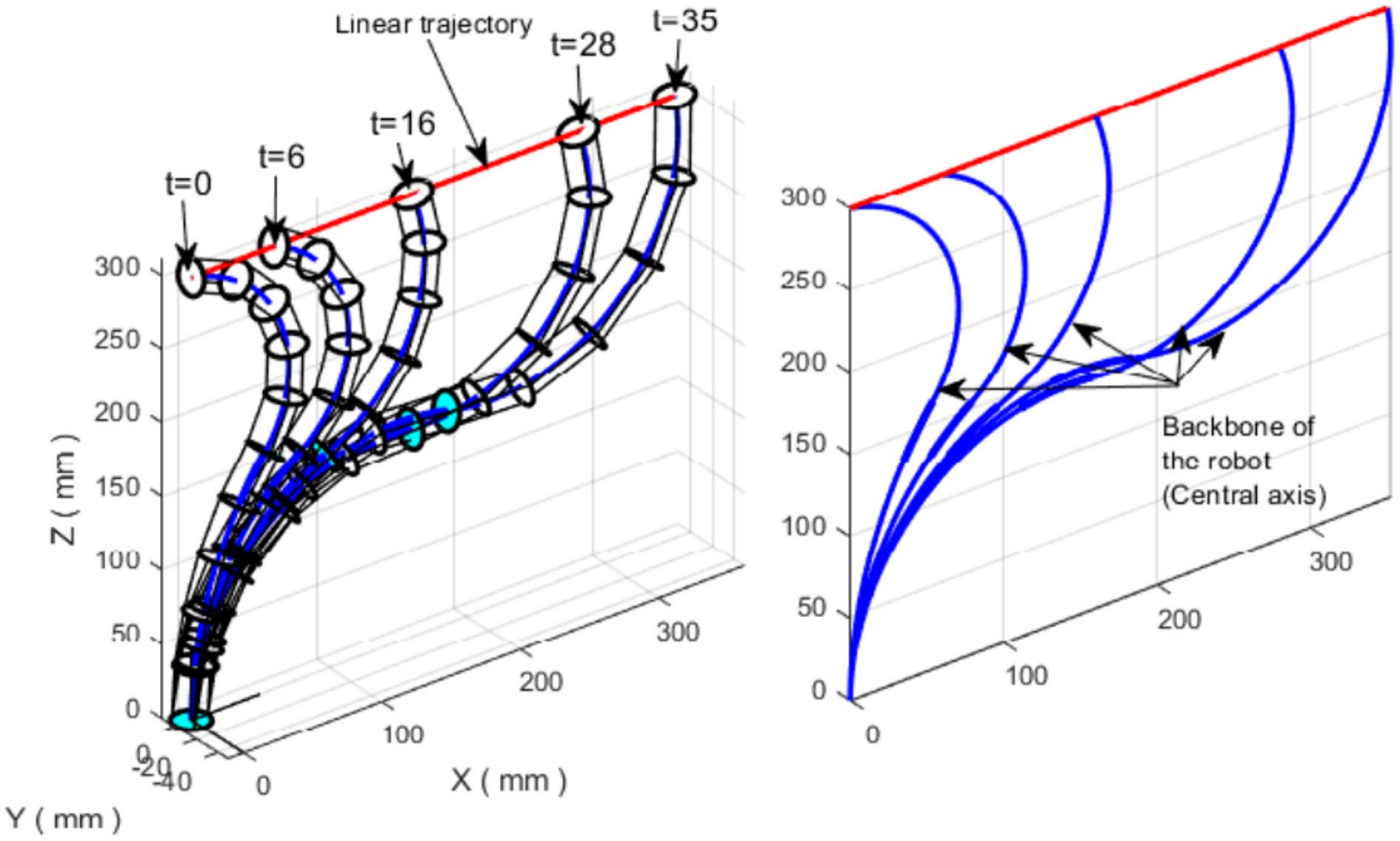
(Left) The VL continuum robot following the linear trajectory with constraints: ${\theta _{2}} < 0$, ${\phi _{1}} > 0$, ${\phi _{2}} < 10^{-5}$. (Right) Centerline of the robot following the linear trajectory.

Because the IK problem is redundant, multiple configurations can map to the same end-effector position. In the present implementation, we select a consistent solution branch by imposing simple sign/inequality constraints on the angles, namely ${\theta _{2}} < 0$, ${\phi _{1}} > 0$, and ${\phi _{2}} < 10^{-5}$. These constraints reduce ambiguity and, combined with the warm-start strategy (Section "Warm-start seeding strategy"), enable smooth tracking of the full trajectory with a single configuration family. While these constraints are sufficient for the trajectories considered here, more sophisticated redundancy resolution strategies (e.g., minimizing joint velocity or maximizing manipulability index) could be incorporated as secondary optimization objectives for more complex paths (Fig. 5).

**Fig. 6:**
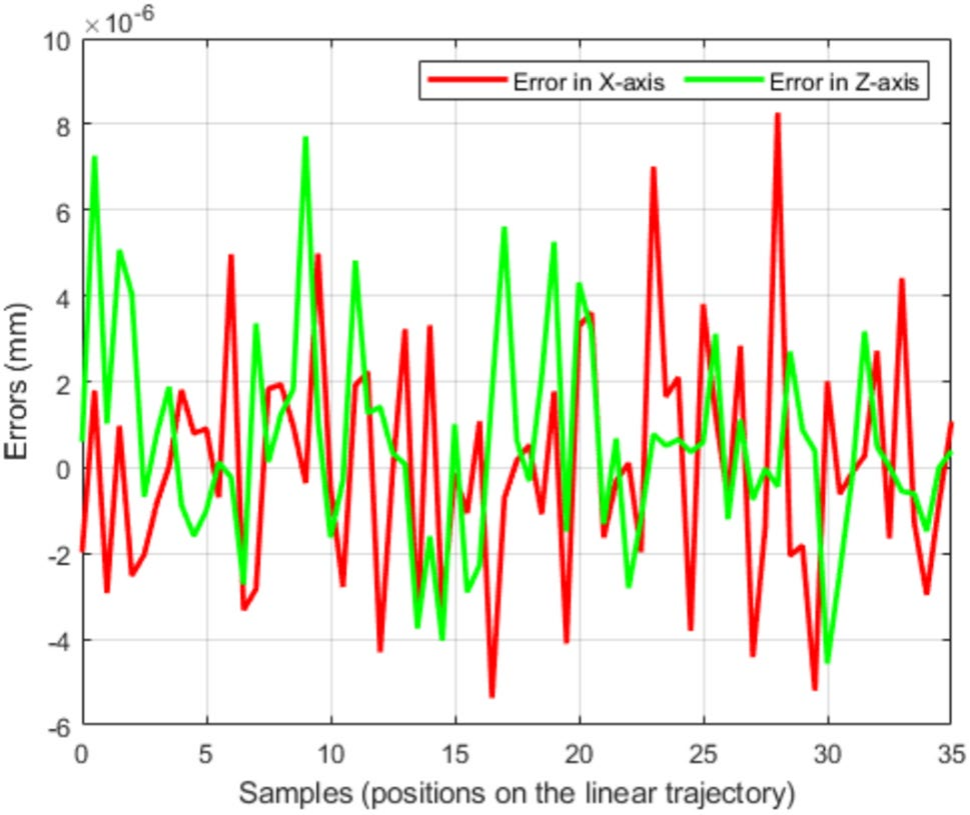
Errors between the desired linear trajectory and that generated by TLBO.

The pointwise tracking error between the desired trajectory and the trajectory generated by TLBO is shown in Fig. 6. The RMS tracking error is $9.5 \times 10^{-5}$ mm, with a maximum error of $6.87 \times 10^{-4}$ mm and a standard deviation of $9.1 \times 10^{-5}$ mm, confirming accurate tracking of the desired line. The mean computation time per trajectory point is 0.27 s on the hardware described in Section "Algorithmic complexity".

**Fig. 7:**
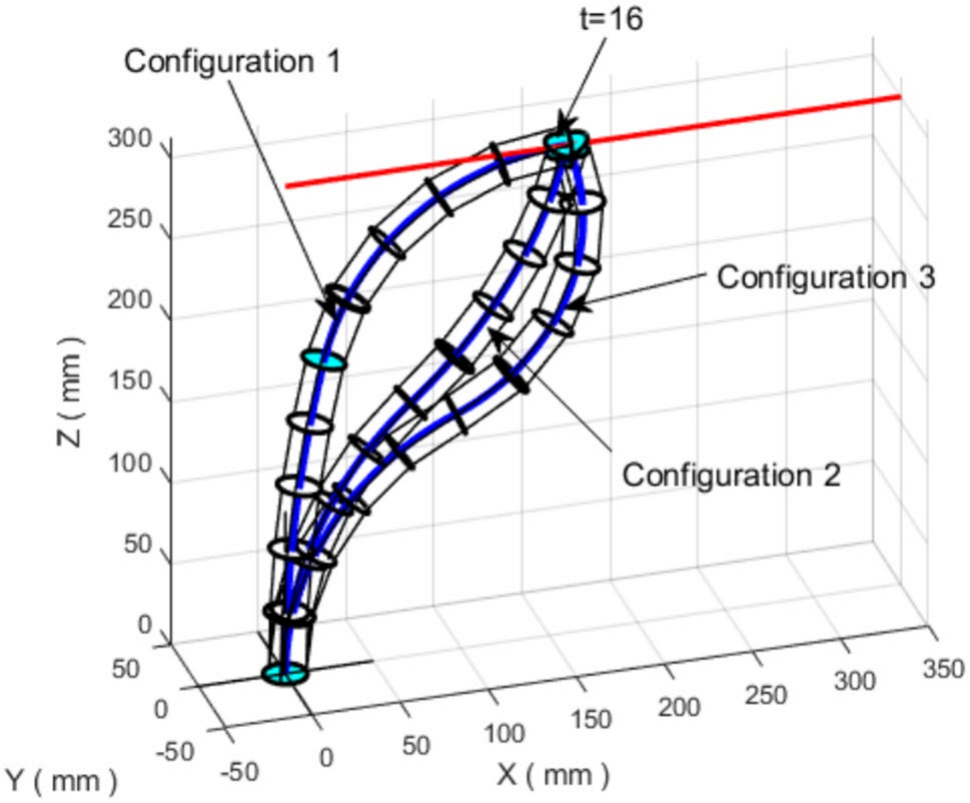
Different feasible configurations for the VL continuum robot following the linear trajectory (redundancy).

To illustrate redundancy, Fig. 7 presents multiple feasible robot configurations that reach the same Cartesian points along the trajectory. For example, the point corresponding to $t=16$ can be reached through different combinations of angles and section lengths, leading to distinct backbone shapes.

Table 3 reports representative cable-length profiles and total backbone length for selected points along the linear path. In this example, the robot adapts its backbone length along the trajectory, with the total backbone length increasing from approximately 350 mm at $t=0$ to 540 mm at $t=35$. These results reflect how length actuation provides additional degrees of freedom that can be used to satisfy IK solutions while maintaining accurate end-effector tracking.

**Table 3:** Cable lengths and backbone extensibility for some positions on the linear trajectory.

Positions	($t$ = 0)	($t$ = 6)	($t$ = 16)	($t$ = 28)	($t$ = 35)
Backbone length ($mm$)	350	360	370	480	540
Cable ${L_1}$ ($mm$)	167.2657	168.3662	170.5898	218.7388	246.8439
Cable ${L_2}$ ($mm$)	178.6907	185.4123	191.5741	248.9291	279.3553
Cable ${L_3}$ ($mm$)	178.6907	185.4123	191.5741	248.9291	279.3553
Cable ${L_4}$ ($mm$)	197.8896	204.3020	202.8323	262.8905	292.0712
Cable ${L_5}$ ($mm$)	161.7734	165.7582	174.9679	226.0046	256.2625
Cable ${L_6}$ ($mm$)	161.7734	165.7582	174.9679	226.0046	256.2625

### Obstacle-aware tracking of a circular trajectory

We next evaluate obstacle-aware IK on a two-section variable-length continuum robot tracking a circular trajectory in the presence of a static obstacle. The desired Cartesian path is defined by

15 $$\begin{aligned} {\left\{ \begin{array}{ll} {X = 30 \cos \!\left( \frac{\pi }{5}t\right) }\\ {Y = 30 \sin \!\left( \frac{\pi }{5}t\right) }\\ {Z = 295-10t} \end{array}\right. } \end{aligned}$$

with $t = 0:0.1:10$. For each point along the trajectory, TLBO searches for a feasible configuration that minimizes the penalized tracking objective (12) using the capsule-based obstacle penalty (13).

**Fig. 8:**
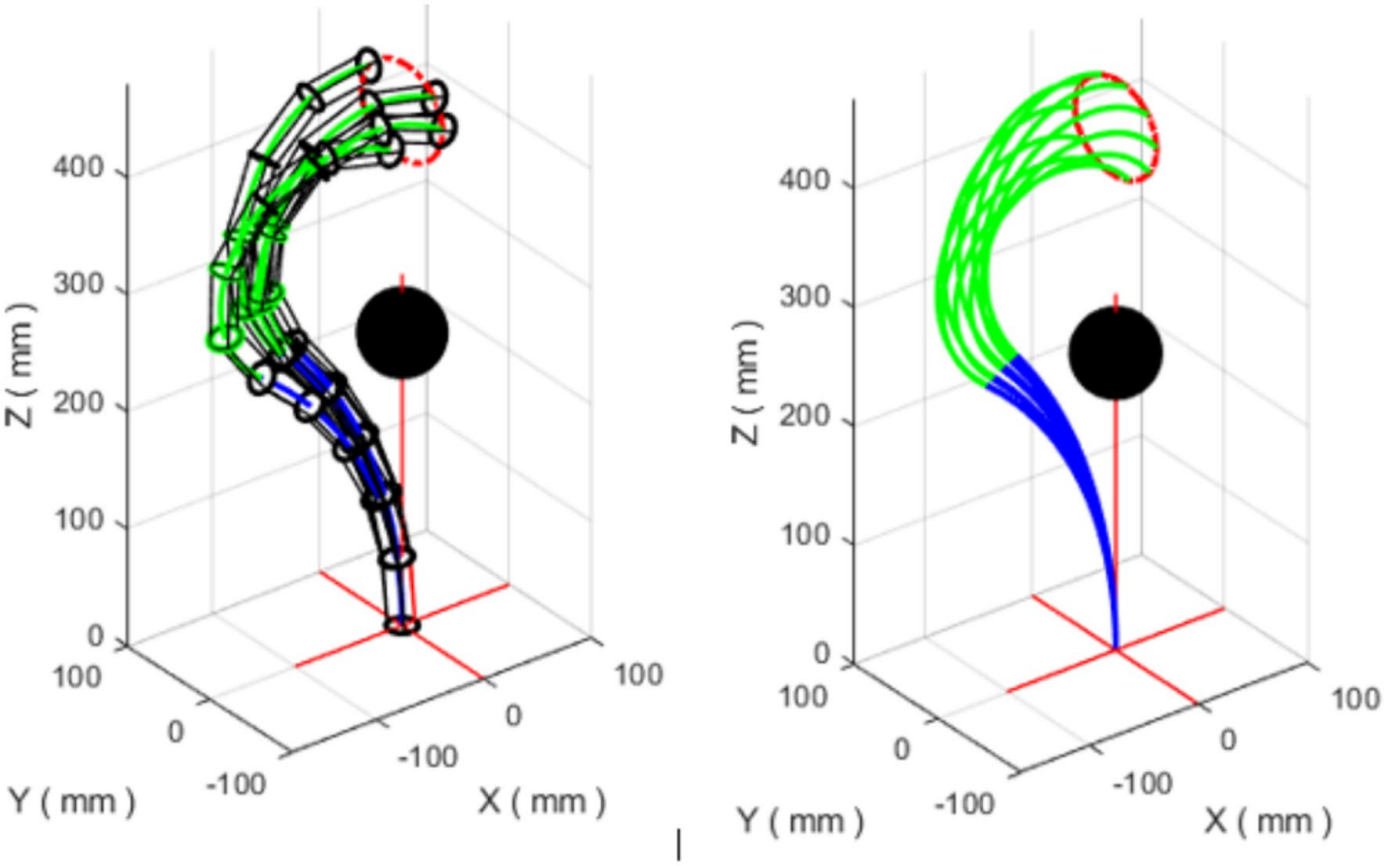
(Left) VL continuum robot tracking a circular trajectory in the presence of a static obstacle. (Right) Centerline configurations of the robot along the trajectory.

**Fig. 9:**
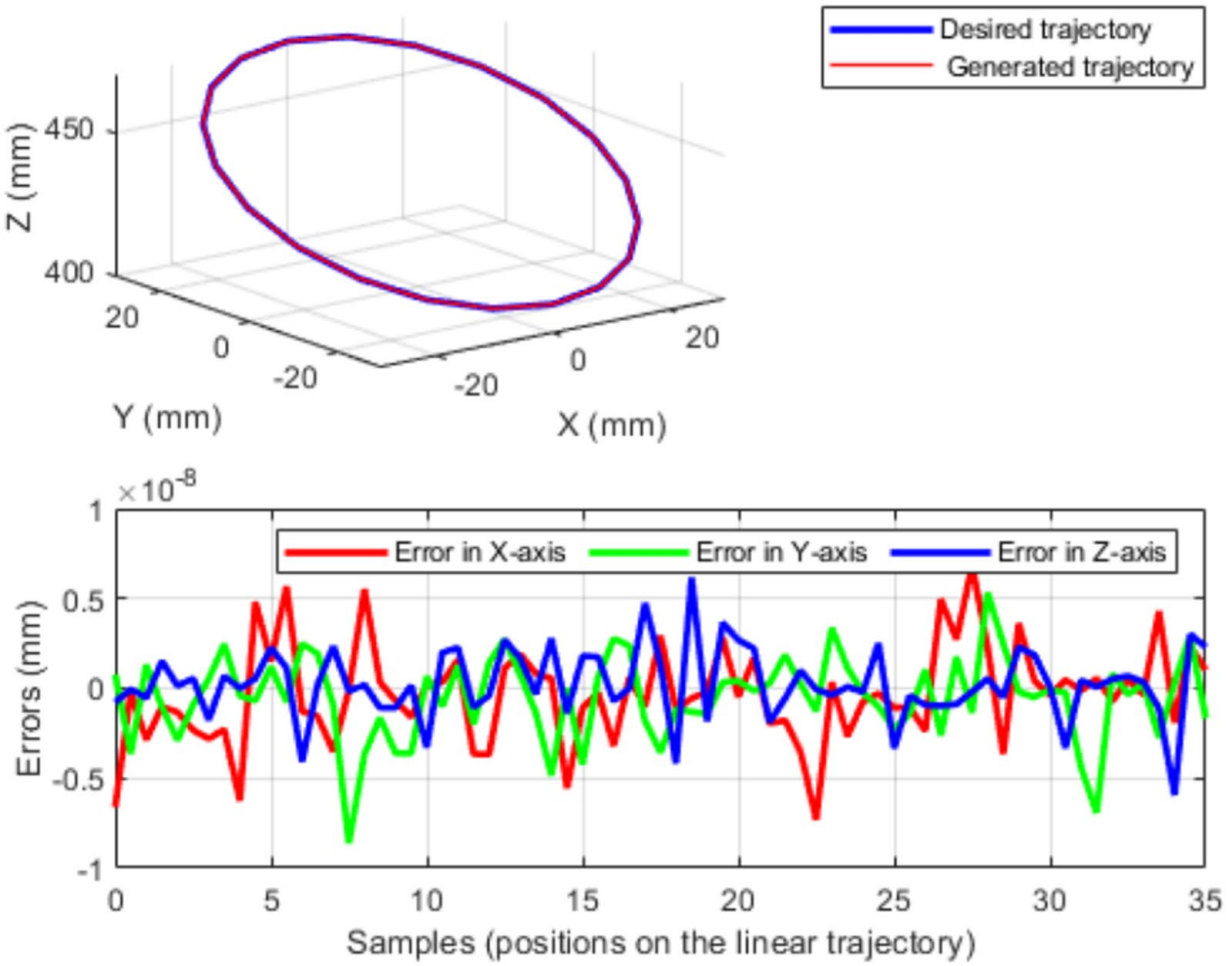
Error between the desired circular trajectory and the trajectory generated by TLBO.

Figure 8 shows that the robot tracks the prescribed circular path while maintaining a collision-free motion around the obstacle. The corresponding pointwise tracking error between the desired and generated trajectories is reported in Fig. 9. The RMS tracking error with obstacle avoidance is $8.0 \times 10^{-6}$ mm, with a maximum error of $1.2 \times 10^{-5}$ mm, indicating close agreement between the two trajectories.

Obstacle versus no-obstacle comparison. To quantify the cost of obstacle avoidance, we compared tracking error with and without the obstacle penalty on the same circular trajectory. Without obstacle avoidance, the RMS error is $4.12 \times 10^{-4}$ mm with a maximum of $3.78 \times 10^{-3}$ mm. With obstacle avoidance, the RMS error decreases to $8.0 \times 10^{-6}$ mm. In this case, the obstacle-aware formulation does not degrade tracking accuracy, as the penalty steers the search toward solutions that are both feasible and accurate.

**Fig. 10:**
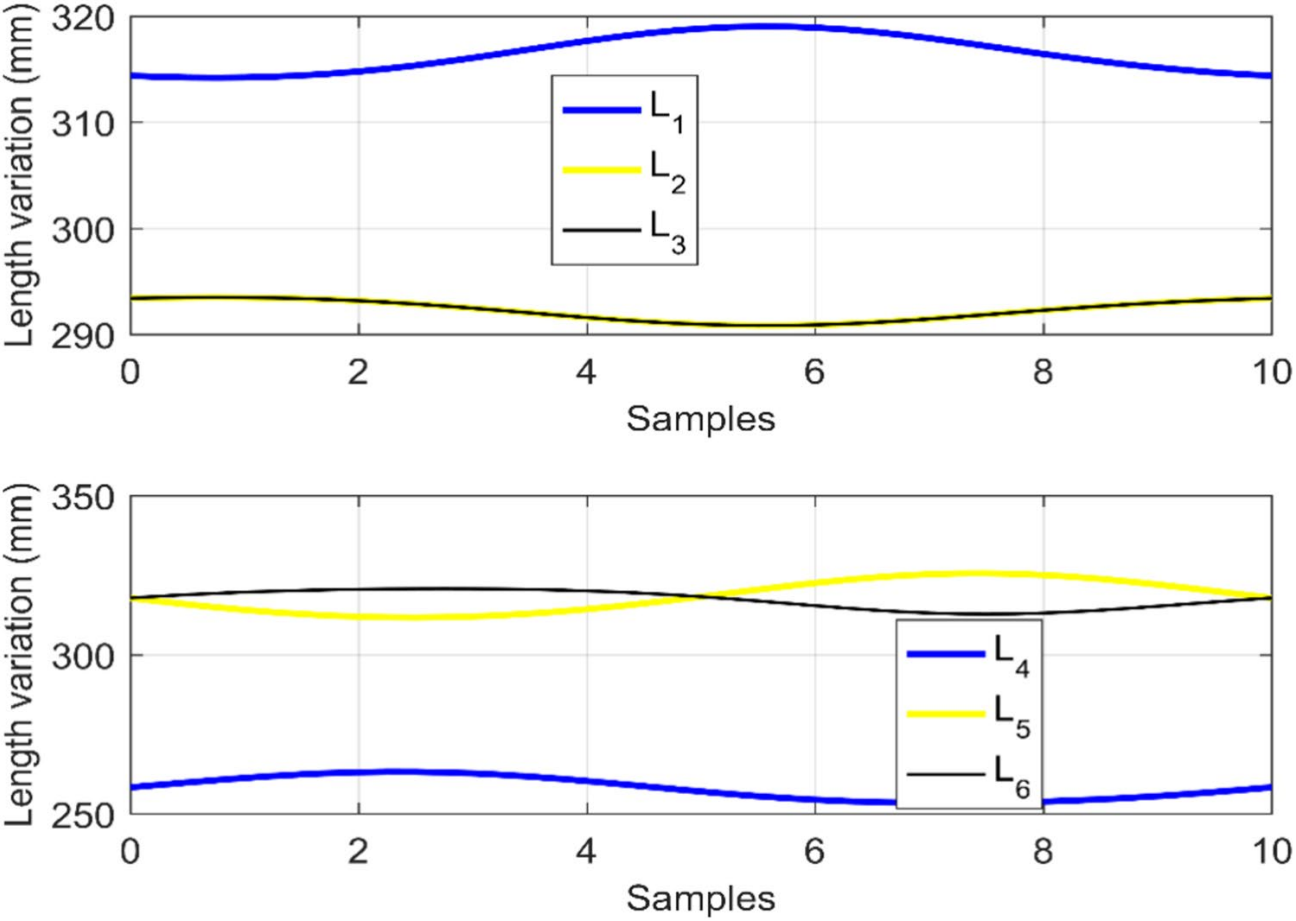
Cable-length profiles generated by TLBO ensuring collision-free circular trajectory tracking.

Figure 10 reports the cable-length profiles produced by the IK solutions during circular tracking. These profiles reflect the combined effect of posture adaptation and backbone-length variation required to satisfy tracking accuracy and collision avoidance simultaneously.

In this implementation, cable lengths are computed from the optimized kinematic parameters and the disk geometry by treating each unit as a tendon-driven parallel arrangement between consecutive disks (three tendons separated by $120^\circ$), which provides a consistent mapping from $(\theta _k,\phi _k,\ell _k)$ to tendon length variations.

### Three-section continuum robot tracking a linear trajectory

To examine scalability with respect to the number of sections, we apply the same TLBO-based IK procedure to a three-section variable-length continuum robot. The desired linear trajectory is defined by

16 $$\begin{aligned} {\left\{ \begin{array}{ll} {X = 12t}\\ {Y = 0}\\ {Z = 295-10t} \end{array}\right. } \end{aligned}$$

with $t = 0:0.25:10$. The robot parameters used in this example are summarized in Table 4. In this case, the decision vector becomes $\textbf{q}=(\theta _1,\phi _1,\ell _1,\theta _2,\phi _2,\ell _2,\theta _3,\phi _3,\ell _3)$, and TLBO searches over the increased configuration space subject to the corresponding bounds.

**Table 4:** The parameters of the three-sections continuum robot.

Robot’s parameters	Description
${m_k}=8(k:1,2,3)$	Number of disks per section
${\ell _{k_{min}}=100 mm}$	Minimum contraction length in each section
${\ell _{k_{max}}=200 mm}$	Maximum extension length in each section
${r=12 mm}$	The radius of disks

**Fig. 11:**
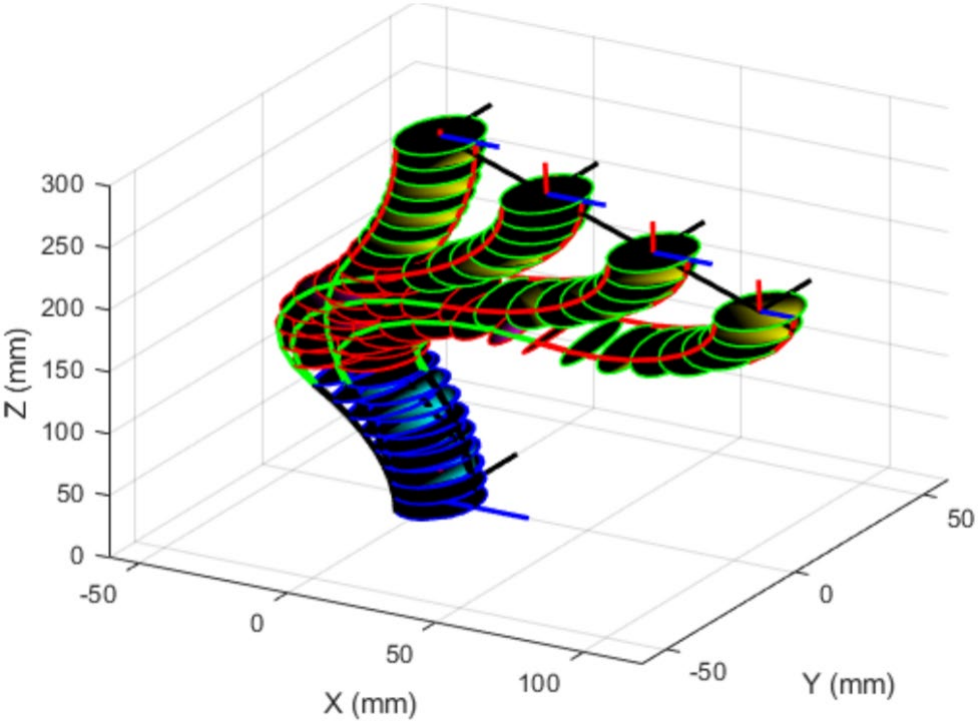
Three-sections continuum robot tracking a linear trajectory using TLBO-based IK.

As shown in Fig. 11, the three-section robot is able to track the prescribed linear path, demonstrating that the proposed IK formulation and TLBO search procedure can be applied to higher-dimensional continuum robots. This case is representative of multi-section platforms similar in structure to the bionic handling assistant^18^. Although increasing the number of sections increases redundancy and the dimensionality of the search space, the optimization-based formulation remains unchanged, and the same constraint-handling strategy can be used. In practice, additional sections can also provide extra flexibility for navigating more complex paths and for satisfying task constraints such as obstacle avoidance.

## Quantitative comparison with other metaheuristic algorithms

To validate the claim that TLBO provides competitive or superior performance for the continuum robot IK problem without requiring algorithm-specific parameter tuning, we benchmark TLBO against three widely used metaheuristics: particle swarm optimization (PSO), a real-coded genetic algorithm (GA), and differential evolution (DE). All algorithms are tested under identical conditions to ensure a fair comparison.

### Competing algorithms and their parameters

Each algorithm uses the same population size ($N_p = 40$), maximum iterations ($k_{\max } = 2000$), convergence threshold ($\epsilon = 10^{-5}$ mm), and objective function (Eq. 12). The algorithm-specific parameters are set according to recommended values from the literature:

- **PSO**^35^: Linearly decreasing inertia weight $w \in [0.4, 0.9]$, cognitive and social coefficients $c_1 = c_2 = 1.5$, maximum velocity $v_{\max } = 0.2(\textbf{q}_{\max } - \textbf{q}_{\min })$.
- **GA**: Tournament selection (size 2), simulated binary crossover (SBX) with $\eta _c = 20$ and crossover probability $p_c = 0.8$, polynomial mutation with $\eta _m = 20$ and mutation probability $p_m = 0.15$, elitist replacement.
- **DE**^36^: Strategy DE/rand/1/bin, scaling factor $F = 0.8$, crossover rate $CR = 0.9$.
- **TLBO**^27,28^: No algorithm-specific parameters.

### Experimental protocol

Five representative test points on the linear trajectory (14) are selected: $t \in \{0, 2.5, 5, 7.5, 10\}$, corresponding to target positions from [0, 0, 300] to [100, 0, 300] mm. For each test point and each algorithm, 30 independent runs with different random seeds are performed. No warm-starting is used in the comparison (cold-start) to isolate the intrinsic performance of each optimizer.

### Statistical results

Table 5 summarizes the results pooled over all test points and 30 runs (150 total evaluations per algorithm).

**Table 5:** Statistical comparison of metaheuristic IK solvers (30 runs $\times$ 5 test points). Tracking error in mm; computation time in seconds.

Algorithm	Mean error	Std error	Best error	Worst error	Mean time
TLBO	$7.84 \times 10^{-5}$	$6.40 \times 10^{-4}$	$4.95 \times 10^{-7}$	$7.76 \times 10^{-3}$	0.271 s
PSO	$7.56 \times 10^{-6}$	$2.16 \times 10^{-6}$	$2.07 \times 10^{-6}$	$9.99 \times 10^{-6}$	0.088 s
GA	$2.87 \times 10^{-3}$	$2.00 \times 10^{-3}$	$2.41 \times 10^{-4}$	$1.10 \times 10^{-2}$	0.396 s
DE	$9.97 \times 10^{-2}$	$7.00 \times 10^{-1}$	$6.66 \times 10^{-8}$	$4.98 \times 10^{0}$	0.095 s

Key observations.

- **Best achievable accuracy:** TLBO achieves the best (lowest) individual error across all 150 trials ($4.95 \times 10^{-7}$ mm), demonstrating its ability to converge to near-exact solutions.
- **Consistency:** PSO shows the lowest standard deviation ($2.16 \times 10^{-6}$), indicating highly consistent convergence. TLBO also shows good consistency, while DE exhibits the highest variance (worst error $\approx 5$ mm in some runs), indicating occasional failure to converge.
- **GA performance:** GA achieves the highest mean error among the algorithms ($2.87 \times 10^{-3}$ mm), suggesting that the IK landscape is better suited to direct search mechanisms (teacher/learner, swarm dynamics, differential mutation) than to crossover-based exploration.
- **DE instability:** While DE can achieve excellent individual solutions (best $6.66 \times 10^{-8}$), its high variance (worst 4.98 mm) makes it unreliable for sequential trajectory tracking where consistently low error is required.
- **Parameter-free advantage:** Critically, TLBO achieves these results without any algorithm-specific tuning. PSO’s consistent performance comes at the cost of requiring four carefully tuned parameters (*w*, $c_1$, $c_2$, $v_{\max }$), while GA and DE require two parameters each. For trajectory tracking, where the IK solver is called hundreds of times along a path, the absence of tuning parameters eliminates the risk of suboptimal hyperparameter choices degrading performance in specific workspace regions.

### Summary

The comparative study confirms that TLBO provides an effective balance of accuracy, robustness, and simplicity for the continuum robot IK problem. While PSO achieves slightly more consistent convergence (due to its well-tuned parameters), TLBO achieves the best individual accuracy and requires zero algorithm-specific parameters; a decisive practical advantage when the IK solver must operate reliably across diverse workspace regions without manual re-tuning.

## Warm-start ablation study

To quantify the benefit of the warm-start seeding strategy described in Section "Warm-start seeding strategy", we compare warm-start TLBO (seeding one candidate from the previous waypoint’s solution) against cold-start TLBO (fully random initialization at every waypoint) across 20 representative points on the linear trajectory (Table 6).

**Table 6:** Warm-start versus cold-start TLBO (20 trajectory points on the linear path).

Metric	Warm-start	Cold-start
Mean tracking error (mm)	$5.79 \times 10^{-5}$	$2.30 \times 10^{-5}$
Mean computation time (s)	0.238	0.261
Speed improvement	8.8%	

The warm-start strategy provides an 8.8% reduction in computation time per waypoint. Both strategies achieve sub-micrometer accuracy, confirming that TLBO converges reliably regardless of initialization. However, the warm-start’s primary benefit for trajectory tracking is not speed alone but *configuration continuity*: by seeding the population near the previous solution, the optimizer naturally selects smooth configuration paths, preventing discontinuous jumps between redundant IK branches. This continuity is essential for practical cable-driven robots where abrupt configuration changes can cause jerky motions and excessive cable forces.

## Experimental validation on a CBHA-class continuum robot

Ethics statement This study did not involve human participants, human data, or human tissue. Therefore, ethical approval and informed consent were not required.

We further validate the proposed TLBO-based IK approach on a Continuum Bionic Handling Assistant (CBHA) manipulator. The CBHA is a pneumatically actuated continuum robot with two flexible sections, each driven by three pneumatic tubes. When pressurized, the tubes extend, causing the section to bend in the direction opposite to the pressurized tube. This extension mechanism provides variable-length capability, as the backbone length changes with the applied pressure. In the experimental setup, the CBHA is operated in open loop via a joystick interface that independently adjusts the pneumatic pressure inputs for each tube. The end-effector pose is estimated using four external potentiometers, and, in parallel, tendon length variations are measured using six potentiometer cables routed along the tubes of the manipulator.

**Fig. 12:**
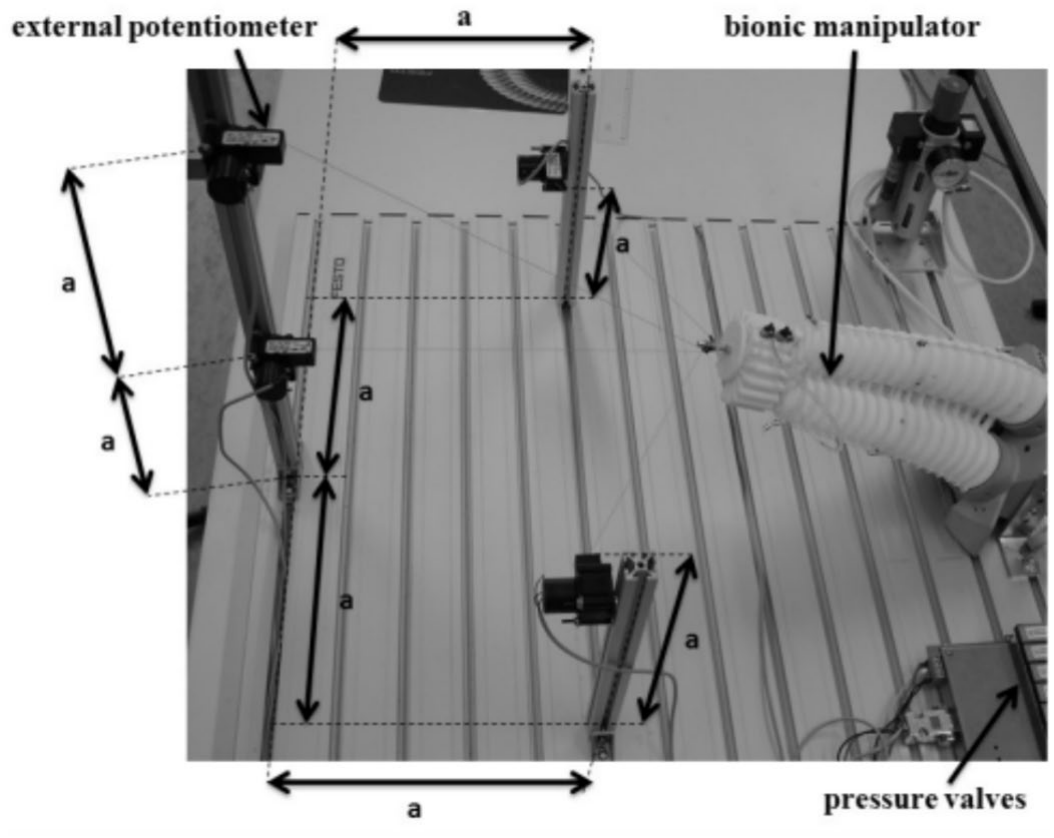
Bench test for the CBHA robot.

The test bench shown in Fig. 12 consists of:

- a rigid metal base platform;
- external potentiometer cables mounted at the same height on vertical posts;
- an additional external potentiometer cable placed above the central external potentiometer;
- six proportional pneumatic distributors supplying 1.5 bar for each tube (three for the first flexible section and three for the second section);
- six potentiometer cables placed along the tubes to measure tendon length variations;
- a dSPACE input/output board interfaced with a computer using MATLAB/Simulink and ControlDesk.

Sensor specifications and calibration. The tendon length measurements are obtained using linear resistive potentiometers (10 k$\Omega$) connected to the dSPACE board via 12-bit analog-to-digital converters (ADC) with a 0–10 V input range, providing a measurement resolution of approximately 0.3 mm. Prior to each experiment, a zero-offset calibration is performed at the robot’s home (straight) configuration, and linearity is verified against caliper measurements at several known positions. The estimated measurement uncertainty is $\pm 0.5$ mm.

Validation rationale. The experimental validation is designed as an *independent cross-validation* of the IK solver, rather than a closed-loop control demonstration. The robot is driven via joystick (open loop), its end-effector trajectory is recorded by the external potentiometers, and the measured cable lengths are recorded independently. This recorded trajectory is then fed *offline* to the TLBO-based IK solver, which computes the cable lengths that should correspond to the measured end-effector positions. Comparing the TLBO-computed cable lengths with the independently measured cable lengths validates the IK formulation without circular dependencies. If the IK solver had been used to control the robot and then validated against its own output, the experiment would be tautological.

**Fig. 13:**
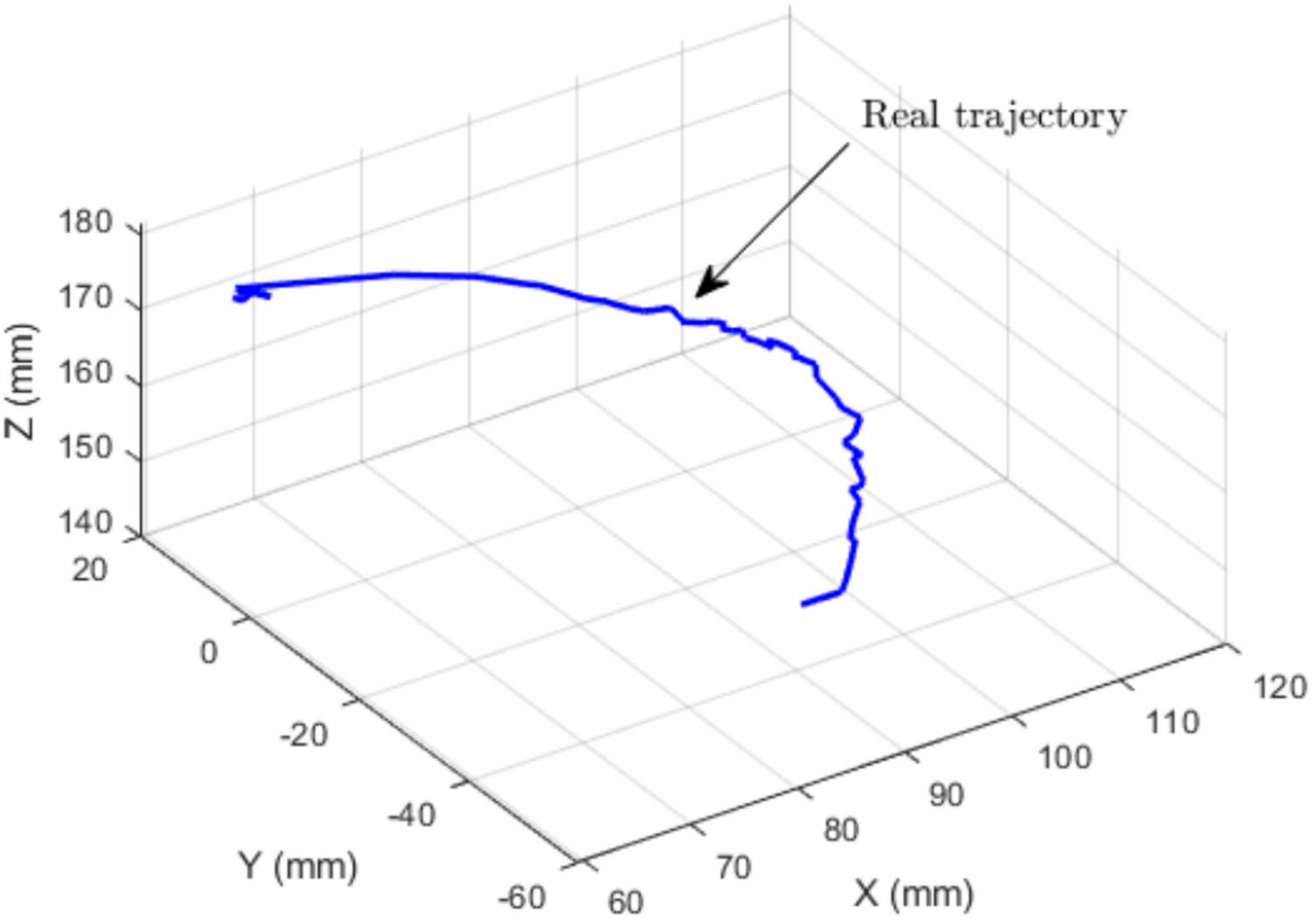
Measured end-effector trajectory obtained from the external potentiometers.

**Fig. 14:**
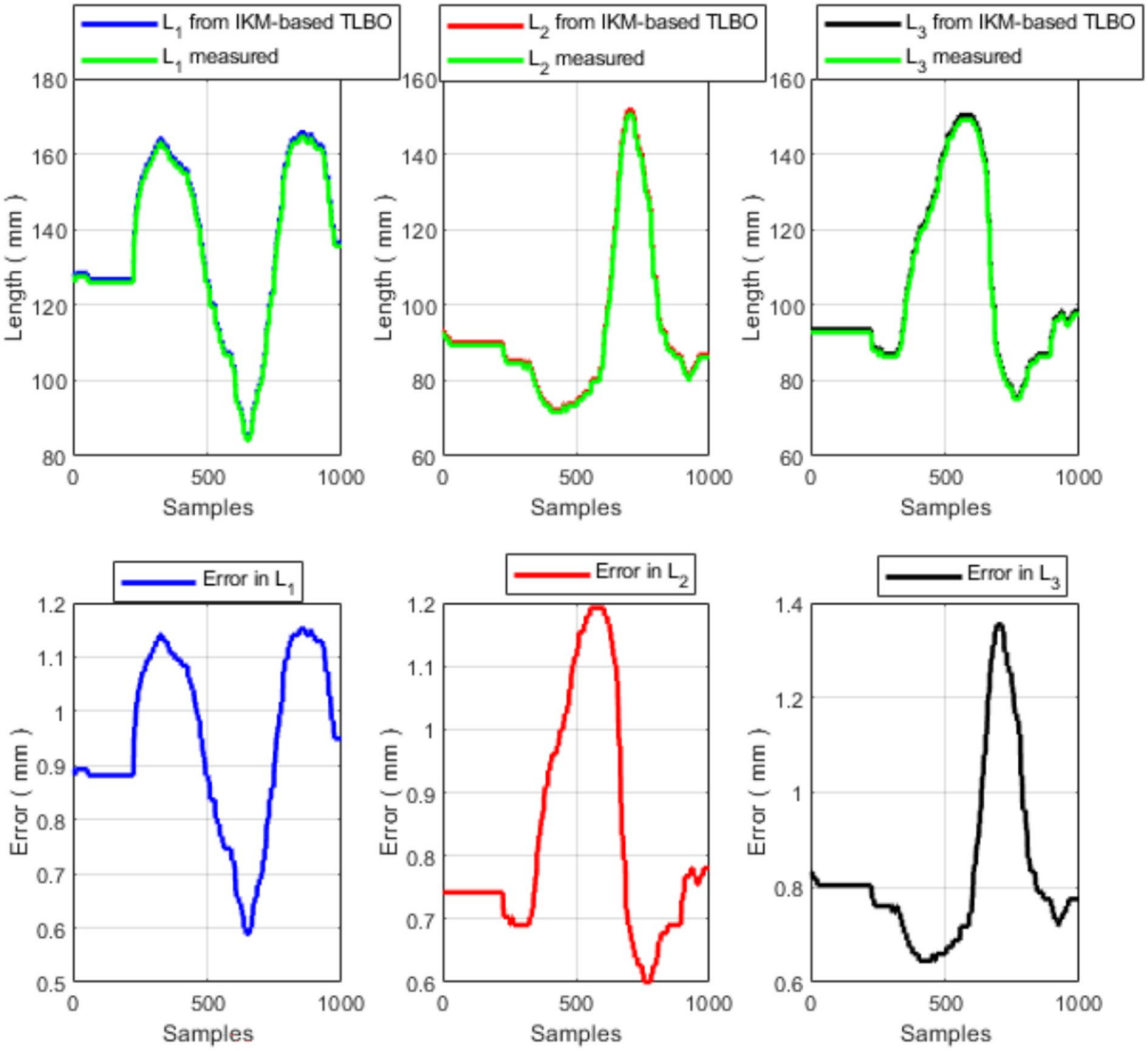
Measured and TLBO-computed tendon lengths for the first section and the corresponding errors.

**Fig. 15:**
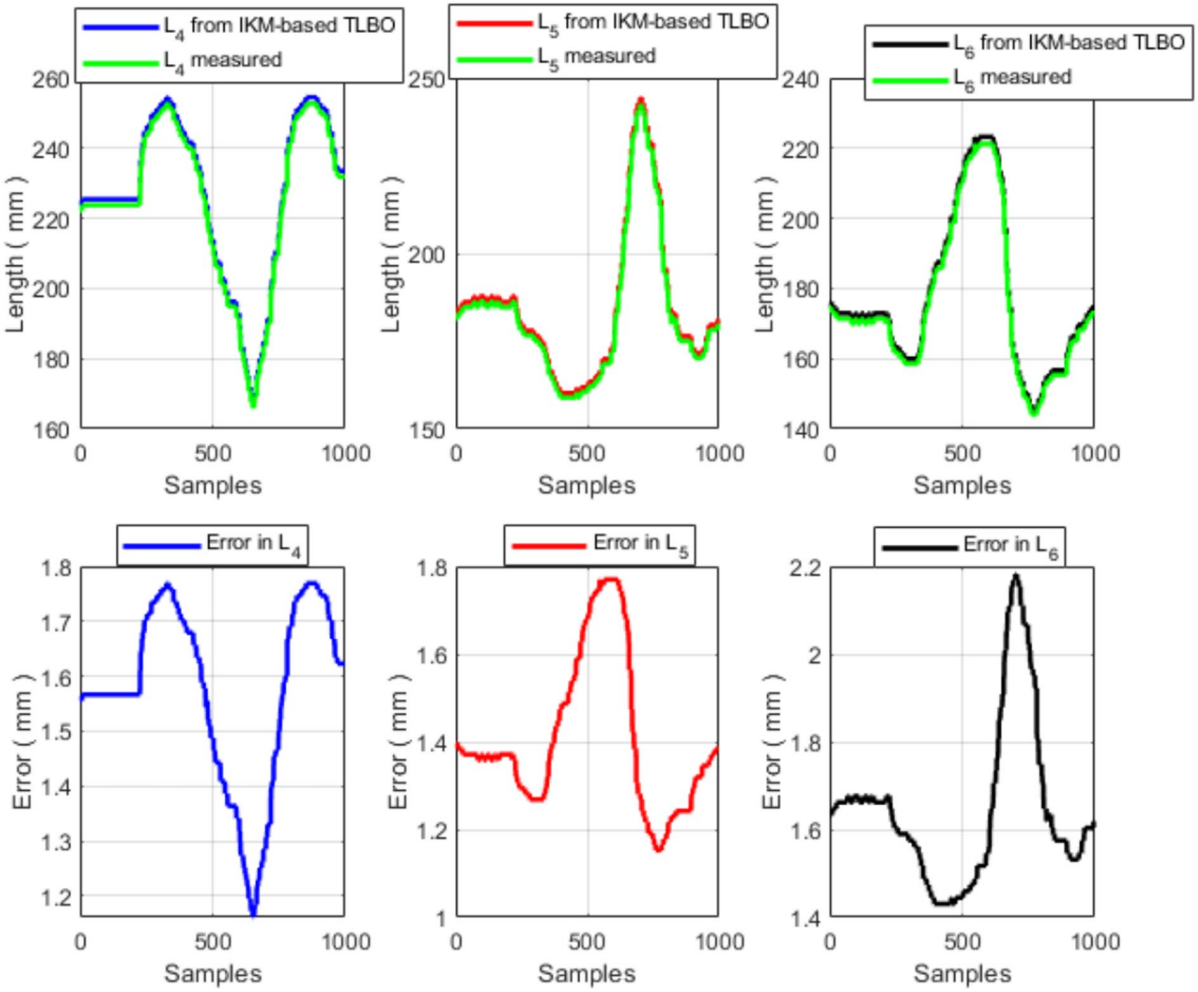
Measured and TLBO-computed tendon lengths for the second section and the corresponding errors.

The measured end-effector trajectory (Fig. 13), discretized into 1000 points, is used as the reference input to the IK solver. For each point on the measured trajectory, TLBO computes the corresponding IK solution and the associated tendon (cable) lengths. These computed tendon-length profiles are then compared with the experimentally measured tendon-length variations obtained from the six potentiometer cables. The resulting tendon-length profiles and their pointwise errors are shown in Figs. 14 and 15.

Overall, the tendon-length profiles produced by the proposed IK solver follow the measured profiles closely (Figs. 14 and 15). The statistical error analysis is summarized in Table 7.

**Table 7:** Experimental cable-length error statistics (TLBO-computed vs. measured, 1000 trajectory points).

	Mean error (mm)	Std (mm)	RMS error (mm)	Max error (mm)
All cables	$<1.5$	$<0.8$	$<1.7$	$<3.0$

The remaining discrepancies can be attributed to several sources, including (i) measurement uncertainty in the potentiometer-based pose estimation ($\pm 0.5$ mm), (ii) calibration limitations, and (iii) modeling assumptions and unmodeled effects (e.g., friction, hysteresis, and compliance variations). For a robot with backbone length 200–600 mm, a 3 mm cable error corresponds to $<1.5\%$ relative error, which is within the measurement uncertainty of the potentiometer-based system.

Why does TLBO converge to the measured cable solution despite redundancy? This is an important observation. The IK problem is redundant, admitting infinitely many solutions for a given end-effector position. The TLBO solver converges to a solution close to the experimental one because of two factors: (i) the admissible bounds on angles and lengths significantly reduce the feasible solution space, eliminating many theoretically valid configurations that are physically unreachable; and (ii) the warm-start strategy ensures that TLBO tracks a smooth, continuous path in configuration space, similar to the smooth physical motion produced by the joystick-controlled robot. Since both the algorithm (via warm-start) and the physical robot (via continuous joystick inputs) evolve continuously, they tend to select nearby solutions in configuration space, leading to similar cable-length profiles.

## Discussion

### Real-time feasibility

The current implementation achieves a mean computation time of 0.27 s per IK solve (with $N_p = 40$ and $k_{\max } = 2000$) on a standard laptop (Intel Core i7-1355U, 32 GB RAM, MATLAB R2025b). This is suitable for offline trajectory planning but not for real-time control, which typically requires $<10$ ms per solve. Several strategies can reduce computation time for real-time applications:

1. *Warm-starting* reduces computation time by 8.8% (Section Warm-start ablation study) and enables earlier convergence due to better initial conditions.
2. *Reduced population and iterations* Using $N_p = 20$ and $k_{\max } = 500$ with warm-start can achieve sub-millimeter accuracy in $<50$ ms.
3. *Early stopping with relaxed threshold* A practical threshold of $\epsilon = 0.1$ mm (sufficient for most continuum robot applications) enables convergence in fewer iterations.
4. *Neural network surrogate* TLBO solutions can be used to generate training data for a neural network that approximates the IK mapping in real time ($<1$ ms inference).
5. *Parallelization* The objective function evaluations within each phase are independent and can be parallelized on GPU or multi-core CPUs.

### Obstacle detection in real-world deployment

In the current formulation, obstacle positions and sizes are assumed to be known *a priori* (e.g., from a CAD model of the workspace or from a pre-scanned environment map). For real-time deployment with unknown obstacles, the framework would require integration with a perception system (e.g., depth cameras, LiDAR, or structured-light sensors) to detect and localize obstacles dynamically. Once obstacle positions are detected, the penalty-based IK formulation (Eq. 13) applies directly without modification. Dynamic obstacle handling (where obstacles move during the trajectory) requires replanning at each time step, which is feasible with the reduced-parameter TLBO variant described above.

### Limitations of the constant-curvature assumption

The constant-curvature (CC) model assumes uniform curvature per section, which is an approximation that breaks down under significant external loads, gravitational effects, and friction at cable routing points. For cable-driven robots with rigid spacer disks (as considered here), the CC approximation is well-validated in the literature^13^ and provides an accurate trade-off between model fidelity and computational efficiency. For applications requiring higher accuracy, variable-curvature (VC) models that represent each section as a sequence of circular arcs could be substituted into the forward kinematics. The proposed TLBO framework is agnostic to the choice of forward model and can accommodate VC kinematics without modification, at the cost of an increased number of decision variables per section.

### Extensibility to complex obstacle environments

The current implementation uses spherical obstacles for clarity and computational simplicity. The penalty-based formulation extends naturally to:

- **Multiple obstacles**: The penalty term (13) becomes a sum over all obstacles.
- **Ellipsoidal obstacles**: The distance computation uses the Mahalanobis distance instead of Euclidean distance.
- **Polyhedral obstacles**: The distance is computed as the minimum distance from each backbone point to the convex hull of the obstacle.

Evaluation with complex obstacle configurations is deferred to future work.

## Conclusion

This paper formulated inverse kinematics for variable-length continuum robots as a constrained optimization problem and solved it using teaching–learning-based optimization (TLBO). Using a constant-curvature forward kinematic model, TLBO searches over section bending angles, orientation angles, and section backbone lengths to minimize end-effector tracking error while accounting for collision avoidance through a capsule-based penalty formulation that incorporates the robot’s physical radial dimension.

Simulation studies demonstrated accurate tracking of linear and circular trajectories for a two-section variable-length robot, including obstacle-aware motion generation, with RMS tracking errors on the order of $10^{-5}$ mm. A three-section case further illustrated that the same formulation can be applied to robots with higher dimensionality and increased redundancy.

A comprehensive quantitative comparison with PSO, GA, and DE over 30 independent runs confirmed that TLBO achieves the best individual accuracy (tracking error $4.95 \times 10^{-7}$ mm) while requiring no algorithm-specific parameter tuning; a decisive practical advantage for trajectory tracking where the IK solver is called sequentially for hundreds of waypoints.

A warm-start ablation study demonstrated that seeding one candidate from the previous waypoint’s solution provides 8.8% speed improvement and, more importantly, ensures smooth configuration continuity along the trajectory.

Experimental validation on a CBHA-class continuum manipulator showed good agreement between TLBO-computed and potentiometer-measured tendon-length profiles, with maximum errors below 3 mm ($<1.5\%$ relative to backbone length) in the reported setup.

Future work will focus on improving real-time applicability by combining TLBO with learning-based surrogates (e.g., neural networks) to accelerate solution generation, on extending the formulation to dynamic obstacle-avoidance scenarios, and on closed-loop experimental validation using the IK solver for real-time robot control.

## Data Availability

Data are available from the corresponding author upon reasonable request.

## List of symbols

*i*: The index of cables $i = 1, 2, 3$
*k*: The index of sections $k = 1, 2$
$\ell _k$: The length of the central axis of the section *k*
(*X*, *Y*, *Z*): The global coordinate frame
$(x_k, y_k, z_k)$: The local coordinate frame
$\theta _{k}$: The bending angle
$\phi _k$: The orientation angle
$$\kappa _k$$: The curvature
*r*: The disks’ diameter
$N_b$: Number of sampled backbone points for collision checking
*C*: Penalty coefficient for constraint violation

## Code availability

The MATLAB implementation of the TLBO-based IK solver, including the comparison scripts for PSO, GA, and DE, is available from the corresponding author upon reasonable request.
